# A report of Kabul internet users on self-medication with over-the-counter medicines

**DOI:** 10.1038/s41598-023-35757-6

**Published:** 2023-05-25

**Authors:** Arash Nemat, Khalid Jan Rezayee, Mohammad Yasir Essar, Wafaa binti Mowlabaccus, Shoaib Ahmad, Mohammad Yousuf Mubarak

**Affiliations:** 1 Department of Microbiology, Kabul University of Medical Sciences, 3rd District, Kabul, 1001 Afghanistan; 2 Department of Global Public Health, Karolinska Institutet, 17177 Solna, Sweden; 3 Department of Medical Laboratory Technology, Kabul University of Medical Sciences, Kabul, Afghanistan; 4 Kabul University of Medical Sciences, Kabul, Afghanistan; 5 Department of Global Health, McMaster University, Hamilton, ON Canada; 6 Department of Medicine, Northampton General Hospital, Northampton, UK; 7 Department of Medicine, District Headquarters Teaching Hospital, Faisalabad, Pakistan

**Keywords:** Health care, Medical research

## Abstract

Self-medication (SM) with over-the-counter (OTC) medications is a prevalent issue in Afghanistan, largely due to poverty, illiteracy, and limited access to healthcare facilities. To better understand the problem, a cross-sectional online survey was conducted using a convenience sampling method based on the availability and accessibility of participants from various parts of the city. Descriptive analysis was used to determine frequency and percentage, and the chi-square test was used to identify any associations. The study found that of the 391 respondents, 75.2% were male, and 69.6% worked in non-health fields. Participants cited cost, convenience, and perceived effectiveness as the main reasons for choosing OTC medications. The study also found that 65.2% of participants had good knowledge of OTC medications, with 96.2% correctly recognizing that OTC medications require a prescription, and 93.6% understanding that long-term use of OTC drugs can have side effects. Educational level and occupation were significantly associated with good knowledge, while only educational level was associated with a good attitude towards OTC medications (p < 0.001). Despite having good knowledge of OTC drugs, participants reported a poor attitude towards their use. Overall, the study highlights the need for greater education and awareness about the appropriate use of OTC medications in Kabul, Afghanistan.

## Introduction

Self-medication (SM) has been described as “the taking of drugs, herbs or home remedies on one's own initiative, or on the advice of another person, without consulting a doctor”^1^. This can include purchasing medications directly from pharmacies, reusing formerly prescribed medicines, or buying over-the-counter (OTC) medications from pharmacies or medical stores^2^. The most commonly self-prescribed medications include analgesics, antipyretics, sedative drugs, specific antibiotics, supplements, herbal medicines, and homeopathic remedies^2^. Adolescent athletes may also consume nutritional supplements like proteins and amino acids, which may complicate the SM phenomenon^3^.

SM is a global issue and may contribute to the human pathogen resistance to antibiotics^1^. One concern about SM is stockpiling, which can lead to a shortage of important drugs that may be needed to treat other serious conditions^4^. Studies conducted on SM shows that it is a very common practice, especially in economically destitute communities^1^. Various factors can lead to the practice of SM, including the need for self-care, sympathy for ailing family members, scarcity of healthcare services, low economic status, ignorance, assumption, excessive advertisements of drugs, and availability of drugs in places other than pharmacies^5^.

The COVID-19 pandemic has exacerbated the issue of SM globally. Fear and anxiety surrounding the pandemic^6^, difficulty in accessing healthcare services due to lockdowns^7^, and misinformation about COVID-19 and potential treatments have all contributed the rise of SM^8^. Countries in sub-Saharan Africa like Togo do not require a prescription for the purchase of antibiotics and have found an increase in the use of SM to prevent against COVID-19. In a cross-sectional study conducted in Lomé, the capital of Togo, a total of 955 participants were questioned and one-third of the individuals in high-risk populations were found to practice SM^9^. Another study in Peru questioned 3,792 study respondents about SM during the pandemic and found majority of respondents practiced SM with acetaminophen for respiratory symptoms mainly because they had a cold or flu^10^. Similarly in Pakistan, the freedom of purchasing pharmaceutical drugs without prescription and the use of unlabeled and unregulated medicinal preparations offered by fake herbalists, homeopaths, and so-called traditional healers has led to misdiagnosis and SM practice^11^. Globally, SM practice with different OTC medications has seen an increase due to the general population struggle against Coronavirus^12^. According to a study, global SM practice with different OTC products and antibiotics has increased from 36.2% in 2019 to 60.4% in 2020^2^.

SM is also a prevalent issue in Afghanistan, as demonstrated by a study of 385 participants who visited community pharmacies. The study found that 73.5% of participants had self-medicated with antibiotics, with the top three being penicillin, metronidazole, and ceftriaxone. The primary reasons for SM with antibiotics included economic problems and a lack of time to visit doctors^13^. Additionally, a study conducted among first- and fifth-year medical students at Kabul University of Medical Sciences found that 25.16% of students reported engaging in SM^14^. While there is evidence of SM in Afghanistan, the body of literature on the subject remains limited. Therefore, there is a need to further study the issue and bring it to the attention of health policymakers and relevant stakeholders.

The current research was unable to use a paper-and-pencil technique with randomly selected samples due to conflict-related limitations. Instead, it intended to raise awareness of the SM among internet users in Afghanistan who use over-the-counter medicines to provide updated insight for researchers to investigate the topic further and inform health policymakers and other relevant stakeholders.

## Results

### Demographics of participants

Based on the responses obtained, it was found that 75.2% of the participants were male. The majority of the participants were over 40 years old (95.1%) and single (65.7%). As for occupation and level of education, more participants were from the non-health staff and university level respectively (69.6%, 92.1%). The majority of participants (69.3%) earned more than $100 per month and most of them (97.4%) funded their health expenses by themselves (Table 1).

**Table 1:** Demographics of participants.

	Categories	Frequency	Percentage
Gender	Male	294	75.2
	Female	94	24.8
Age	> 40 years	372	95.1
	< 40 years	19	4.9
Marital status	Single	257	65.7
	Married	134	34.1
Occupation	Non-health staff	272	69.6
	Health staff	119	30.4
Level of education	Pre-university level	30	7.7
	University level	361	92.1
Income per month	≤ $100	120	30.7
	> $100	271	69.3
Health expenses	Self	381	97.4
	Government or private company health insurance	10	2.6

### Attitude towards over-the-counter medication

Analyzing the attitude toward OTC medication among the participants, it was found that more than 50% believed that OTC is cheap 80.1% and appropriate to treat minor ailments (75.2%). The average response of participants is 5 over 9 with 53.5% having a good attitude. Based on the threshold cut off number, we assumed 5 score to be considered as good attitude among participants (Table 2).

**Table 2:** Attitude towards over-the-counter medication.

Statement		Frequency	Percentage
I use OTC because of doctors’ high visit cost	True	222	56.8
	False	169	43.2
OTC is cheap and available	True	313	80.1
	False	78	19.9
It is appropriate to treat minor ailments with OTC	True	294	75.2
	False	97	24.8
With pharmacist consultation, I feel relax	True	260	66.5
	False	131	33.5
I use OTC because it is convenient to use	True	220	56.3
	False	171	43.7
I use OTC to save time	True	183	46.8
	False	208	53.2
The main cause of OTC use is advertising	True	238	60.9
	False	153	39.1
It’s ok to share OTC with others	True	84	21.5
	False	307	78.5
OTC drugs are not affected by storage conditions, like temperature, moisture, and direct sunlight	True	85	21.7
	False	306	78.3
Total	Median, IQR, range	5	3(0–9)
	Good attitude	209	53.5
	Poor attitude	182	46.5

### Knowledge about over-the-counter medication

Assessing the knowledge of the participants with regards to OTC medications, it was found that more than 50% used OTC because they knew what medications can be used to treat themselves (52.4%) and that they read the leaflets before using 62.7%. Moreover, over 90% of respondents agreed that OTC needs prescription and that long-term use of OTC can have side effects (93.6%). The average response of OTC was 5 out of 9 and 65.2% participants were perceived as having good knowledge. Thus, we considered 5 score as good knowledge among participants (Table 3).

**Table 3:** Knowledge about over-the-counter medication.

Statement		Frequency	Percentage
I use OTC because I know how to treat myself	True	205	52.4
	False	186	47.6
I read medication leaflet before using any drug	True	245	62.7
	False	146	37.3
I know the drug needs prescription	True	376	96.2
	False	15	3.8
Use of herbal products has no harm	True	185	47.3
	False	206	52.7
Long term use of OTC drugs can cause serious side-effect	True	366	93.6
	False	25	6.4
(If suspected side-effects are seen, then one should Immediately stop using the drug	True	248	63.5
	False	143	36.4
If suspected side-effects are seen, take low dose until side-effects subside	True	47	12.0
	False	344	88.0
If suspected side-effects are seen, continue taking the drug regardless of the side-effects	True	22	5.6
	False	369	94.4
Total	Median, IQR, range	5	2 (1–9)
	Good knowledge	255	65.2
	Poor knowledge	136	34.8

### Comparison between participants having good or poor knowledge of OTC

Participants with a good knowledge of OTC were significantly from a higher level of education and were health staff (p-value < 0.05) (Table 4).

**Table 4:** Comparison between participants having good or poor knowledge of OTC.

Categories		Good knowledge (n,%)		Poor knowledge (n,%)		P value
Gender	Male	189	35.7	105	64.3	0.501
	Female	66	68.0	31	32.0	
Age	> 40 years	239	64.2	133	35.8	0.075
	< 40 years	16	84.2	3	15.8	
Marital status	Single	155	60.3	102	39.7	0.531
	Married	100	74.6	34	25.4	
Occupation	Non-health staff	19	63.3	11	36.7	**0.001**
	Health staff	236	65.4	125	34.6	
Level of education	Pre-university level	182	66.9	90	33.1	p < 0.001
	University level	73	61.3	46	38.7	
Income per month	≤ $100	76	63.3	44	36.7	0.603
	> $100	179	66.1	92	33.9	
Health expenses	Self	249	65.4	132	34.6	0.726
	Government or private company health insurance	6	60.0	4	40.0	

Significant values are given in bold.

### Comparison between participants having good or poor attitude of OTC

Comparing those who had good attitudes against those having poor attitudes, it was found that those from a higher education level had a statistically better attitude as compared to those having a lower level of education. (p value < 0.05) (Table 5).

**Table 5:** Comparison between participants having good or poor attitude of OTC.

Categories		Good attitude, N	%	Poor attitude, N	%	P value
Gender	Male	160	54.4	134	45.6	0.504
	Female	48	49.5	49	50.5	
Age	> 40 years	8	42.1	174	46.8	0.691
	< 40 years	11	57.9	198	53.2	
Marital status	Single	135	52.5	122	47.5	0.612
	Married	74	55.2	60	44.8	
Occupation	Non-health staff	16	53.3	14	46.7	0.989
	Health staff	193	46.5	168	53.5	
Level of education	Pre-university level	162	59.6	110	40.4	p < 0.001
	University level	72	60.5	47	39.5	
Income per month	≤ $100	67	55.8	53	44.2	0.530
	> $100	142	52.4	129	47.6	
Health expenses	Self	203	53.3	178	46.7	0.674
	Government or private company health insurance	6	60.0	4	40.0	

## Discussion

Afghanistan is a country grappling with numerous challenges, including a long history of conflict and an economy heavily reliant on foreign aid^15^. The country's healthcare system is strained and lacks sufficient resources, leading many people resorting to SM with OTC medications. The quality of education in Afghanistan is poor, further exacerbating the cycle of poverty. To improve the standard of living in Afghanistan, it is essential to have a responsive government committed to the well-being of its citizens and the nation as a whole, as well as skilled professionals capable of effectively managing the country's challenges. In addition, cooperation with donors can help address the significant gaps in education, economics, and health in Afghanistan.

Before the pandemic, SM was already a significant public health concern in Afghanistan, and the pandemic has only exacerbated the situation, introducing new challenges. In the present study, 65.2% of the participants reported a good knowledge about the use of OTC medications, with those who had a higher level of education reporting even better knowledge. This could be attributed to the multiple studies conducted on SM in Afghanistan, which may have contributed to increased awareness among people and policy implementation^13,14^. This finding is similar to that reported in Jordan, where a majority of the participants reported good knowledge about the OTC products^16^. In this study, participants with a higher education degree reported good knowledge. However, in a study conducted in Saudi Arabia, 69.7% of the participants reported poor knowledge, and higher education and employment were found to be statistically significant factors^17^.

In the present study, more than 90% of respondents agreed that OTC medication needs prescription (96.2%) and that long-term use of OTC can result in side effects. This is similar with a study conducted in Saudi Arabia where the majority of participants reported that analgesic is accompanied by side effects. In this study, participants believed that the causes of analgesic are the result of misuse of the analgesic which are easily obtained without prescription^18^.

Regarding attitude, nearly half (46.5%) of the participants in this study reported having poor attitude towards OTC medications. This finding is consistent with a study conducted in Asmara, Eritrea, where 44.7% of the participants continued to take drugs despite experiencing health problems^19^. This could be attributed to socio-economic factors such as poverty, accessibility to healthcare resources, which may impact the attitude of participants.

One reason for SM is for treatment of minor illness. In the present study, 75.2% of the participants self-medicated for this reason. Similar findings have been reported in studies conducted in Eritrea and Thailand. Other reasons for SM included easy access to drugs and cost savings^19,20^.

The present study reveals that more than 56.8% of the participants used OTC medications because they found the doctors’ fee quite high. In a poor country like Afghanistan, where half of the population lives below poverty line^16^, it is not surprising that many people cannot afford doctors’ fees and thus seek alternative methods, such as using OTC medications without a prescription. This finding is similar to what has been reported in India, where participants with poor access to healthcare services were more likely to self-medicate and not consult with medical practitioners and use OTC medications^21^.

In terms of education and financial stability, those participants with a higher level of education and good financial stability reported good knowledge and attitude towards OTC medications. Whereas participants with low level of knowledge and low economic status reported poor attitude and knowledge towards OTC medications. These findings are consistent with those of studies conducted in Greece and Saudi Arabia^17,22^.

### Strengthens and limitations

There are several strengths and limitations to our study. One significant strength was that it was conducted during the pandemic, providing an up-to date understanding of self-medication practices in the country. Additionally, our study identified a significant number of participants who still practice SM, which could be useful for policymakers in implementing timely policies to prevent unnecessary self-medication.

However, the study had several limitations as well. Firstly, it was conducted online, which excluded participants without internet access and may have introduced participation bias toward those with social media access, such as Facebook and WhatsApp. As a result, the study’s findings could be subject to bias. Secondly, the study had a narrow focus, employing a simple questionnaire and only involving the capital city of Kabul. Further research is necessary to examine this topic in other parts of the country. Moreover, the study’s results were affected by an uneven distribution of male participants and university students, which may not accurately represent the population parameters of Kabul. Therefore, more research is needed to determine if this skewed representation is due to the fact that internet and social media usage is more prevalent among university students and graduates. We hope that research institutions, organizations and health authorities who have funding will conduct more in-depth, nationwide research on this issue to gain a better understanding of self-medication practices in Afghanistan.

## Conclusion

This study concluded that participants had good knowledge about the use of OTC drugs, but a poor attitude was reported towards them. Therefore, it is recommended that policymakers work towards improving participant’s attitude by implementing new policies and campaigns. In addition, research from other parts of the country is also required to provide a better understanding of the use of OTC drugs in Afghanistan.

## Methods

### Study population and data collection

An online cross-sectional survey was conducted in Kabul, Afghanistan to assess the knowledge and attitudes towards the use of over-the-counter medication (OTC) among the general public who had access to the internet. The survey was conducted from July to November 2021 through a Google Form link shared via social media platforms such as Facebook (Meta), WhatsApp, Facebook Messenger, and Telegram. The questionnaire included a brief background, objective, strategy, voluntary nature of participation, declaration of anonymity and secrecy, instructions for filling out and its submission.

Data were collected online through crowd-sourced convenience sampling based on the availability and accessibility of participants from various parts of the city. The questionnaire was created in English and then translated into Dari, widely spoken in Afghanistan, and verified using face and content validity approaches. Content validity was performed by the expert researchers to ensure the survey was representative of the construct it was intended to assess. Face validity was determined through pilot testing, and changes were made to the questionnaire as needed.

Before completing the questionnaire, participants' were asked for their consent, and 391 (294 male and 97 female) individuals from both rural and urban areas of Kabul were included. Kabul is the capital city of Afghanistan and is diverse and densely populated. The city has become overcrowded due to political upheaval and a lack of opportunity in other parts of the country. The inclusion criteria for this study were adults aged 18 or older living in Kabul who had internet and social media access, regardless of education level or profession, and who agreed to participate voluntarily. Exclusion criteria included individuals under the age of 18 and those living temporarily in Kabul. Sure, we were asked in the survey where people live and exclude those who didn't live at the time of survey. Therefore, the study primarily focused on adult residents of Kabul to determine their attitudes and responses towards consuming drugs without a prescription. A pilot study was conducted to assess the feasibility of the research approach and to confirm that the questionnaire was properly prepared to acquire all the information required. To eliminate uncertainty, the original questionnaire was updated, and repetitive items were removed.

### Sampling method

The sample size required for the study was calculated using Krejcie and Morgan’s table. Considering the total number of internet users in Afghanistan 7,337,489 or total population of Kabul city 4,400,000 with 99% CI and 5% margin of error, the sample size calculated was 385^23^.

### Statistical analysis

The data were collected and entered into a Microsoft Excel spreadsheet and then entered into the Statistical Package for Social Sciences (SPSS) version 23 for analysis. Simple descriptive analysis was computed for demographic characteristics, and the rest of items were explained in frequency and percentage.

### Ethical statement

The study was approved by the Ethics Committee of Microbiology Department of Kabul University of Medical Sciences (Approval code: KUMS/ RECMD-193). All processes were performed in accordance with relevant guidelines and regulations, including the declaration of Helsinki and subsequent revisions.

### Consent to participate

All study participants provided their informed consents to participate in the study before completing the survey.

## Data Availability

Data cannot be shared publicly because of ethical restriction and respect for anonymity. Data are available upon request from Dr. Arash Nemat, Academic member of Microbiology Department, Kabul University of Medical Sciences via (dr.arashnemat@yahoo.com).
